# College and Hospital Notes

**Published:** 1904-07

**Authors:** 


### COLLEGE AND HOSPITAL NOTES.

---

The Frederick Ferris Thompson Memorial Hospital, at Canandaigua, was dedicated June 14, 1904, with appropriate ceremonies. The Journal acknowledges the courtesy of an invitation to attend and regrets its inability to accept.

---

Dr. Walter Lindley, of Los Angeles, the editor of the *Southern California Practitioner,* has been elected dean of the Medical

College of the University of Southern California, which is about to begin its 20th session. Dr. Lindley was one of the organisers of the school and is professor of gynecology in that institution.

---

Dr. ALBERT VANDER VEER has resigned as dean of the Albany Medical College, an office he has held for many years. This action was rendered necessary by a recent law which prevents a regent of the University of the State of New York acting as dean of a medical college. Dr. Samuel B. Ward has been chosen to fill the vacancy caused by Dr. Vander Veer's resignation.

